# Understanding and Managing Immune-Related Adverse Events Associated With Immune Checkpoint Inhibitors in Patients With Advanced Melanoma

**DOI:** 10.6004/jadpro.2017.8.1.5

**Published:** 2017-01-01

**Authors:** Alyona Weinstein, Ruth-Ann Gordon, Mary Kate Kasler, Matthew Burke, Smita Ranjan, Jackie Hodgetts, Vanessa Reed, Yelena Shames, Nana Prempeh-Keteku, Karla Lingard

**Affiliations:** 1 Memorial Sloan Kettering Cancer Center, New York, New York, USA;; 2 Yale University School of Medicine, New Haven, Connecticut, USA;; 3 University of Louisville, Louisville, Kentucky, USA;; 4 Christie Hospital, Manchester, UK;; 5 Royal Marsden Hospital, London, UK

## Abstract

The immune checkpoint inhibitors ipilimumab, nivolumab, and pembrolizumab represent a substantial improvement in treating advanced melanoma but are associated with adverse events (AEs) likely related to general immunologic enhancement. To ensure that patients receive optimal benefit from these agents, prompt assessment and treatment of AEs are essential. We review the efficacy and safety profiles of these immune checkpoint inhibitors and describe guidelines for managing immune-related AEs. We also present case studies describing the management of toxicities in patients receiving immune checkpoint inhibitor therapy. These cases illustrate the importance of collecting a detailed medical history when administering immunotherapy, as this information is necessary to establish baseline, inform monitoring, and determine the etiology of symptoms. Advanced practice nurses and physician assistants are uniquely positioned to educate patients on the early recognition of AEs and have an important role in establishing appropriate monitoring and open dialogue among services.

The projected incidence of melanoma within the United States is about 87,110 new cases for 2017, with an estimated 9,730 deaths from the disease in the same year (American Cancer Society, 2017). The lifetime risk of developing melanoma is 2.1%, or 1 out of every 50 men and women (National Cancer Institute [NCI], 2016).

Genetic predisposition and environmental stressors contribute to the development of melanoma. Ultraviolet (UV) solar radiation promotes melanoma development through direct mutagenic effects on DNA, production of growth factors, decrease of skin immunity, and promotion of reactive oxygen species, which cause DNA damage (Miller & Mihm, 2006). Normally, melanocytes in the skin respond to UV exposure by stimulating the production of melanin, which then absorbs and dissipates UV radiation. In fair-skinned people, susceptibility to melanoma can occur as a result of genetic impairments in the production of melanin. For example, as many as 40% of hereditary melanomas can be linked to germline mutations in the cyclin-dependent kinase inhibitor 2A (CDKN2A) gene (Miller & Mihm, 2006).

Although most patients with melanoma are diagnosed in the earlier stages (localized, 84%; regional, 9%; distant metastases, 4%), 5-year survival rates based on data from 2005 to 2011 demonstrate considerably worse prognoses for patients diagnosed with metastatic disease (16.6%) compared with those diagnosed at the localized stage (98.3%; NCI, 2016). Therefore, the use of new agents such as immune checkpoint inhibitors in advanced melanoma has been an area of clinical research.

## IMMUNE CHECKPOINT INHIBITION FOR THE TREATMENT OF CANCER

The immune system is able to recognize and mount an immune response against antigenic molecules. However, tumors have developed survival mechanisms for evading immune surveillance, including the use of pathways that normally control immune tolerance (Pardoll, 2012). Two important immune checkpoint pathways are those mediated by cytotoxic T-lymphocyte–associated antigen 4 (CTLA-4) and programmed cell death protein 1 (PD-1). Once activated, T cells upregulate CTLA-4, which can lead to dampening of the immune response early in the activation phase (Hoos et al., 2010). In contrast, PD-1 functions at the later effector phase, playing a role in moderating T-cell activity in peripheral tissues. Immune checkpoint inhibitors have been developed to exploit these CTLA-4 and PD-1 homeostatic controls, blocking events that suppress T-cell activation and allowing T cells to generate sustained antitumor immune responses (Figure; Rothschild, Thommen, Moersig, Müller, & Zippelius, 2015). The mechanism of action of immune checkpoint inhibitors accounts for their efficacy but also for the immune-related adverse events (irAEs) associated with these therapies.

**Figure:**
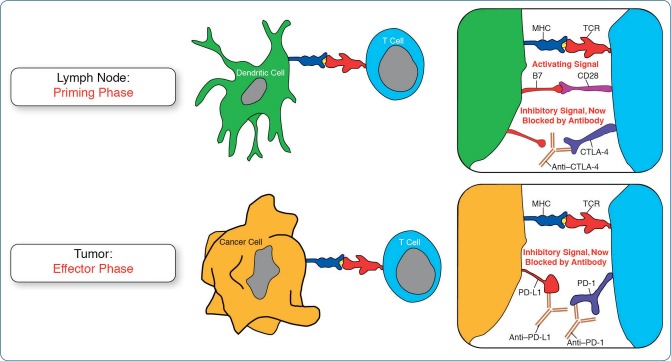
Immune checkpoint inhibition of CTLA-4 or PD-1 pathways by antitumor immunotherapy. CTLA- 4 = cytotoxic T-lymphocyte–associated protein 4; PD-1 = programmed cell death protein 1; MHC = major histocompatibility complex; TCR = T-cell receptor; PD-L1 = programmed cell death ligand 1. Adapted from Rothschild et al. (2015) with permission from EMH Swiss Medical Publishers Ltd.

Currently, three monoclonal antibody immune checkpoint inhibitors have been approved by the US Food and Drug Administration (FDA): the anti–CTLA-4 agent ipilimumab (Yervoy) and the anti–PD-1 agents pembrolizumab (Keytruda) and nivolumab (Opdivo). Ipilimumab was approved in March 2011 for the treatment of unresectable or metastatic melanoma after demonstrating improved overall survival (OS) vs. gp100 peptide vaccine in patients with previously treated metastatic melanoma in a randomized phase III trial (Hodi et al., 2010). Pembrolizumab and nivolumab were initially approved in September and December 2014, respectively, in patients with pretreated unresectable or metastatic melanoma based on tumor response and response duration data in phase I and phase III trials, respectively (Robert et al., 2014; Weber et al., 2015a). More recently, both pembrolizumab and nivolumab received approvals for first-line use in unresectable or metastatic melanoma based on data from phase III trials (Robert et al., 2015a; Robert et al., 2015b). Monotherapy of all three agents is also approved in the European Union. Furthermore, the combination of nivolumab and ipilimumab in the first-line setting is approved by the FDA for the treatment of patients with unresectable or metastatic melanoma based on data from both phase II (Postow et al., 2015) and phase III (Larkin et al., 2015a) trials.

Key efficacy data for these agents as monotherapy and in combination regimens are presented in Table 1. Although ipilimumab is also approved for adjuvant treatment of patients with cutaneous melanoma (Bristol-Myers Squibb, 2015c), and nivolumab and pembrolizumab have indications in metastatic non–small cell lung cancer (Bristol-Myers Squibb, 2016; Merck, 2015a) and renal cell carcinoma (Bristol-Myers Squibb, 2016), our discussion will focus on the use of these agents in advanced melanoma.

**Table 1:**
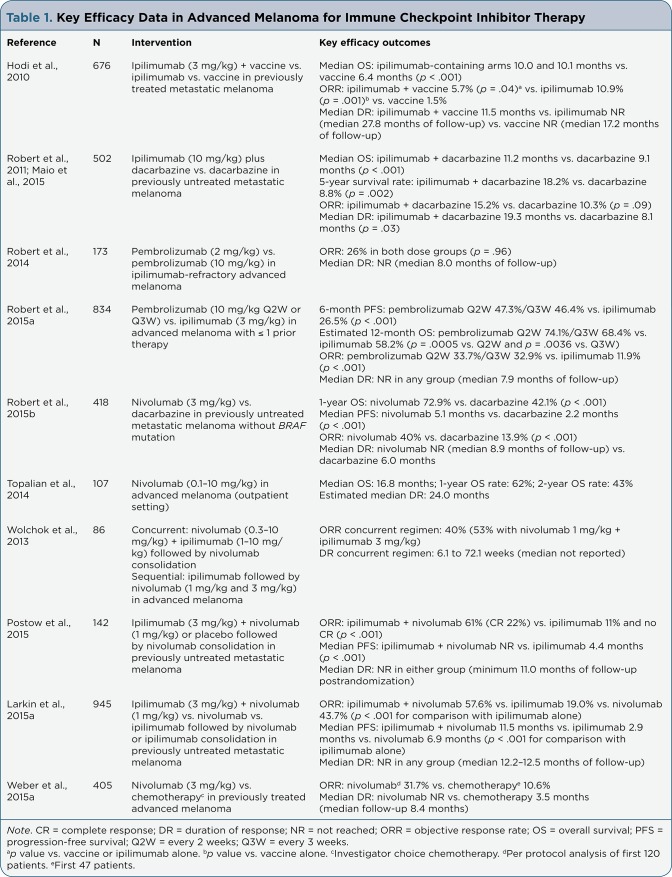
Key Efficacy Data in Advanced Melanoma for Immune Checkpoint Inhibitor Therapy

## EFFICACY OF IMMUNE CHECKPOINT INHIBITORS

In advanced melanoma, objective response rates (ORRs) have ranged from 10.9% to 19.0% for ipilimumab monotherapy, from 26.0% to 33.7% for pembrolizumab monotherapy, and from 31.7% to 43.7% for nivolumab monotherapy, suggesting higher ORR with PD-1 blockade vs. CTLA-4 blockade (Hodi et al., 2010; Robert et al., 2011; Maio et al., 2015; Robert et al., 2014, 2015a, 2015b; Topalian et al., 2014; Wolchok et al., 2013; Postow et al., 2015; Larkin et al., 2015a; Weber et al., 2015a). For example, the phase III KEYNOTE-006 trial comparing pembrolizumab with ipilimumab found a response rate of 33% to 34% (depending on the regimen) with the anti–PD-1 antibody, vs. 12% with ipilimumab (Robert et al., 2015a). Responses across trials have been durable, with median durations of response not reached in most studies.

A median OS of 10 months was reported for ipilimumab in the registrational phase III study (Hodi et al., 2010), and a pooled analysis of 1,861 patients across ipilimumab studies demonstrated a median OS of 11.4 months, suggesting that the durable responses observed with immune checkpoint inhibitors may translate into a survival benefit (Schadendorf et al., 2015). Although median OS has not been reached in phase III studies of PD-1 agents, KEYNOTE-006 found estimated 12-month OS rates of 68% to 74% with pembrolizumab and 58% with ipilimumab (Robert et al., 2015a). Similarly, in the phase III CheckMate 066 trial, the 12-month OS rate was 73% with nivolumab and 42% with dacarbazine (Robert et al., 2015b).

CTLA-4 and PD-1 have distinct but complementary roles in mediating T-cell immune responses at early and later phases of T-cell activation, respectively (Hoos et al., 2010; Rothschild et al., 2015), and dual blockade results in greater antitumor activity than inhibition of either pathway alone in preclinical models (Curran et al., 2010; Selby et al., 2013). These observations have provided a strong rationale for clinical investigation of dual blockade using combined therapy with anti–CTLA-4 and anti–PD-1 antibodies.

The safety and efficacy of ipilimumab and nivolumab given either concurrently or sequentially have been assessed in the phase I CA209-004 study (Wolchok et al., 2013), whereas concurrent therapy has been compared with ipilimumab alone in the phase II CheckMate 069 study (Postow et al., 2015) and with either ipilimumab or nivolumab monotherapy in the phase III CheckMate 067 study (Larkin et al., 2015a). Overall, combination treatment has confirmed preclinical observations and achieved higher ORR (40% to 60%) vs. monotherapy (Table 1).

## TREATMENT-RELATED ADVERSE EVENTS WITH IMMUNOLOGIC ETIOLOGY

Much of the insight into irAEs associated with immune checkpoint inhibitor therapy comes from experience with the anti–CTLA-4 antibody ipili-mumab. Current evidence indicates that this knowledge can be broadly transferred to inhibitors of the PD-1 pathway, with some differences in incidence and severity (Luke & Ott, 2015; Callahan & Wolchok, 2013). The autoimmune basis of irAEs means that any organ system can be affected, but the most common irAEs are dermatologic (rash, pruritus, vitiligo), gastrointestinal (GI; diarrhea, colitis), and endocrine (hypophysitis, hypothyroidism, thyroiditis, adrenal insufficiency; Callahan & Wolchok, 2013).

The irAEs observed in key registrational studies for immune checkpoint inhibitor therapy are summarized in Table 2. Among monotherapies, rates of all-grade rash have been similar across agents. For example, in the phase III KEYNOTE-006 trial comparing pembrolizumab and ipilimumab (Robert et al., 2015a) and in CheckMate 067, which included ipilimumab and nivolumab arms (Larkin et al., 2015a), rates of all-grade diarrhea or colitis were more frequent with anti–CTLA-4 blockade compared with PD-1 pathway–targeted agents. Hypothyroidism appeared more frequently with anti–PD-1 agents vs. ipilimumab (Robert et al., 2015a; Larkin et al., 2015a). The irAEs observed with combination anti–CTLA-4 and anti–PD-1 therapy have generally been similar to those associated with monotherapy, but with a higher frequency (Larkin et al., 2015a).

**Table 2:**
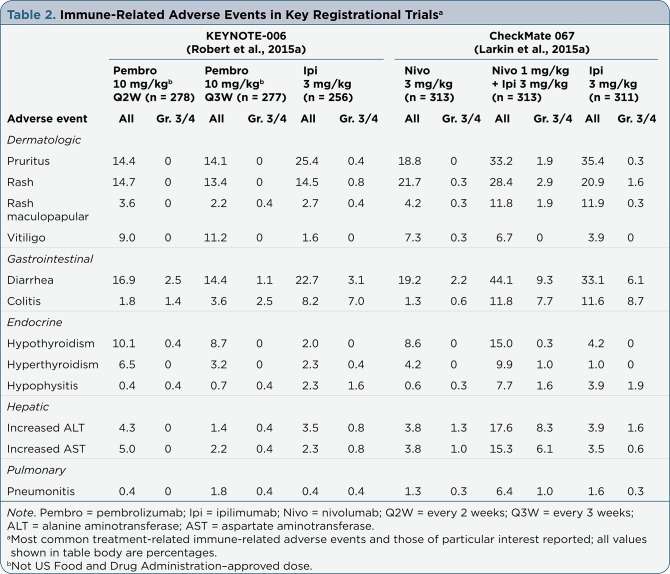
Immune-Related Adverse Events in Key Registrational Trials

The time to onset of irAEs differs according to organ system. Median times to onset and resolution are shown in Table 3. With ipilimumab, dermatologic irAEs occur within 2 to 3 weeks of treatment, followed by GI irAEs within 6 to 8 weeks and endocrine irAEs after around 7 weeks (Weber, et al., 2013; Weber, Kähler, & Hauschild, 2012). Similarly, dermatologic irAEs occur within a median of 5 weeks of treatment with nivolumab, followed by GI irAEs (median, 7 weeks), pulmonary irAEs (median, 9 weeks), and endocrine irAEs (median, 10 weeks; Weber et al., 2015b). Timing of irAEs with the nivolumab and ipilimumab combination has been similar to that seen with monotherapy (Larkin et al., 2015a). For pembrolizumab, median time to onset was 6.5 months for colitis, 1.5 to 3.5 months for endocrine irAEs, and 5 months for pneumonitis (Merck, 2015a).

**Table 3:**
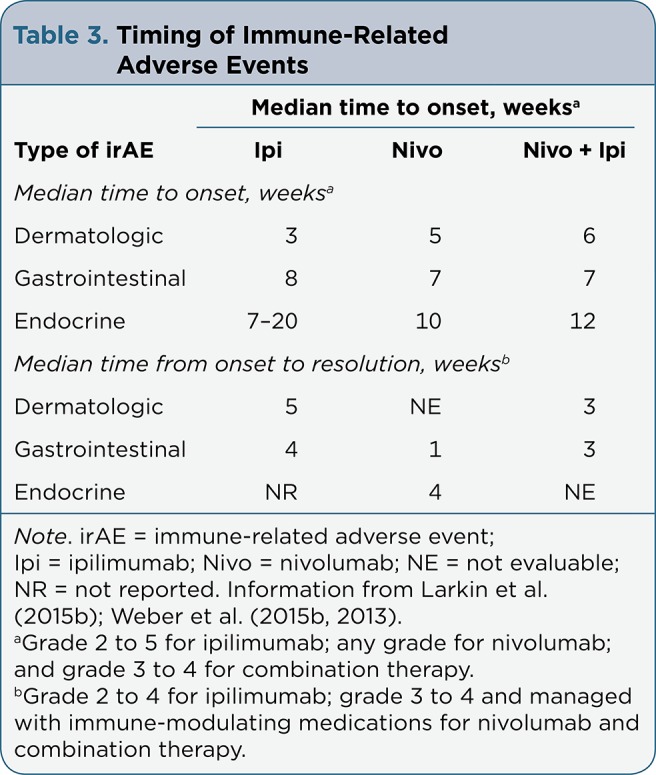
Timing of Immune-Related Adverse Events

Immune-modulating medications such as corticosteroids and antihistamines are often indicated for the management of irAEs. For example, most patients in the combination arms of CheckMate 069 and 067 required either topical or systemic immune-modulating agents to manage irAEs (89% and 83% of patients, respectively), and most severe irAEs resolved when immune-modulating agents were used, except in the case of endocrinopathies (Postow et al., 2015; Larkin et al., 2015a). In CheckMate 067, resolution rates for grade 3/4 irAEs were between 85% and 100% with the nivolumab and ipilimumab regimen for most organ categories, and the median time to resolution ranged from approximately 2 to 4 weeks (Larkin et al., 2015a).

Immune-modulating medications are believed to quell inflammation without interfering with the antitumor response (Postow, 2015). For example, an analysis of a phase III ipilimumab trial found no difference in response between patients receiving vs. not receiving steroids before response (Baurain et al., 2012). Similarly, a pooled analysis of four nivolumab clinical studies found no impact of the use of systemic immune-modulating medications on objective response (Weber et al., 2015b).

## IDENTIFICATION, GRADING, AND MANAGEMENT OF SELECT IRAES

**Dermatologic, GI, and Endocrine**

Across trials of immune checkpoint inhibitors as either monotherapy or combination therapy, dermatologic, GI, and endocrine irAEs have been observed most frequently. Guidance and recommendations on the management of irAEs associated with FDA-approved immune checkpoint inhibitor therapies emphasize the need for prompt identification and intervention (National Comprehensive Cancer Network [NCCN], 2015; Bristol-Myers Squibb, 2016, 2015c; Merck, 2015a). A Risk Evaluation and Mitigation Strategy (REMS) was originally established for ipilimumab to provide guidance for identification and management of irAEs (Bristol-Myers Squibb, 2012); guidelines are still available, although REMS is no longer required (Bartell et al., 2015).

Although no formal REMS programs were required for anti–PD-1 agents, manufacturers have developed additional guidance on irAE identification and management that generally reflect that given for anti–CTLA-4 therapy (Bristol-Myers Squibb, 2016; Merck, 2015a). Table 4 summarizes the signs and symptoms of dermatologic, GI, and endocrine irAEs associated with immune checkpoint blockade (Bristol-Myers Squibb, 2015d, 2015a; Merck, 2015b). The first steps in the management of irAEs are correct identification and grading, and irAEs can be graded according to the Common Terminology Criteria for Adverse Events (CTCAE; Appendix A; NCI, 2009).

**Table 4:**
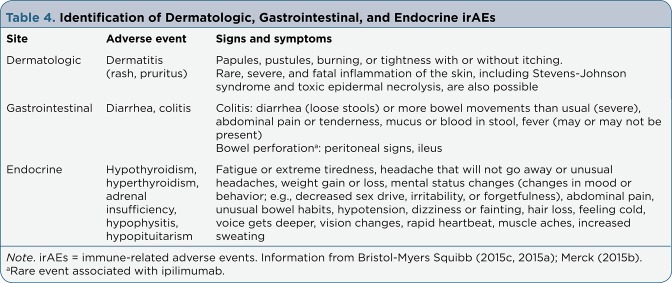
Identification of Dermatologic, Gastrointestinal, and Endocrine irAEs

Guidelines for the management of dermatologic, GI, and endocrine irAEs are summarized in Appendix B. This includes treatment recommendations for moderate or severe AEs, usually requiring treatment interruption and the use of corticosteroid immunosuppression. Of note, endocrine irAEs such as hypophysitis may require lifelong hormonal replacement. Adrenal insufficiency, which can be primary or occur secondary to hypophysitis, requires intense education on appropriate adjustment of steroids to avoid adrenal crisis.

**Appendix A:**
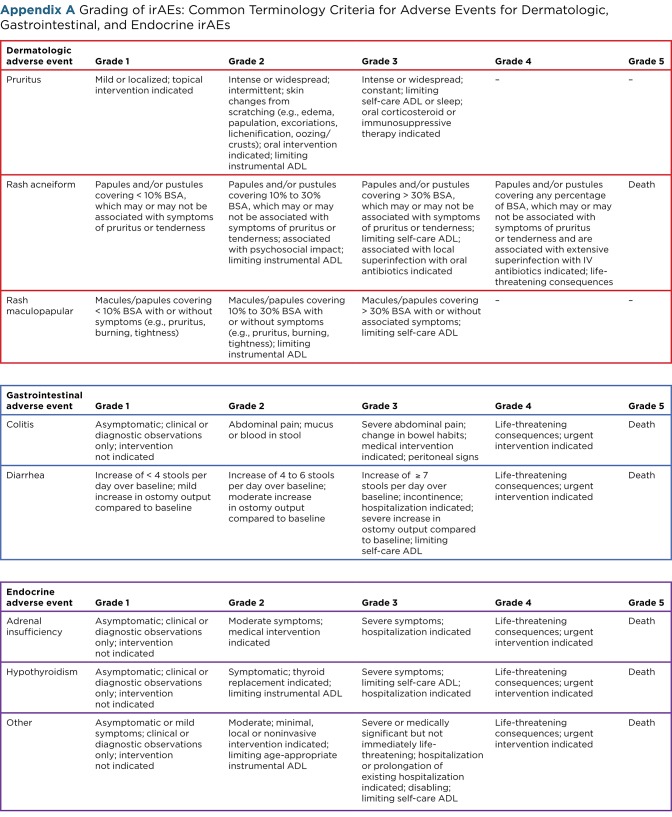
Appendix A Grading of irAEs: Common Terminology Criteria for Adverse Events for Dermatologic, Gastrointestinal, and Endocrine irAEs

**Appendix B:**
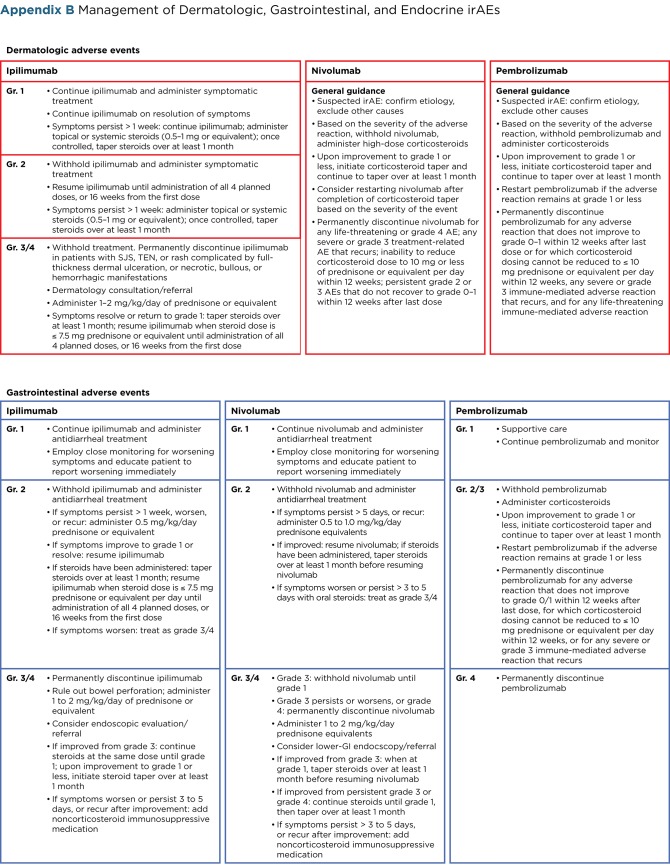
Appendix B Management of Dermatologic, Gastrointestinal, and Endocrine irAEs

**Appendix B:**
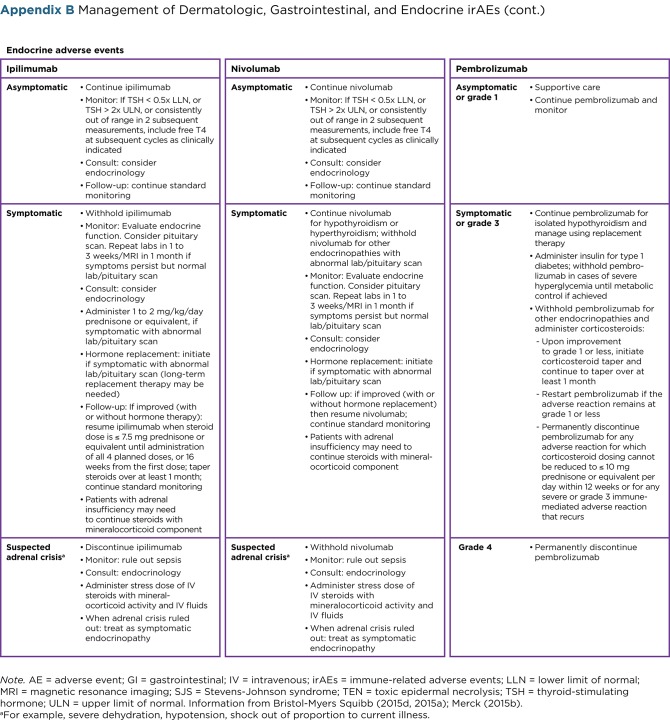
Appendix B Management of Dermatologic, Gastrointestinal, and Endocrine irAEs (cont.)

Recommendations for restarting checkpoint inhibitor therapy and referral points depending on the grade of the irAE are also given. Generally, therapy should be permanently discontinued for severe irAEs, whereas dose is withheld for moderate irAEs (except endocrine AEs) until return to baseline, improvement to mild severity, or complete resolution. Systemic (high-dose) corticosteroids are administered for severe, persistent, or recurring irAEs (Bristol-Myers Squibb, 2015d, 2015a, 2015c, 2016; Merck, 2015b, 2015a). Although rates of irAEs may be numerically higher with combined CTLA-4/PD-1 blockade, no new safety signals have been reported in the phase III CheckMate 067 trial (Larkin et al., 2015a), and management strategies developed for monotherapy remain pertinent in the combination setting. A practical checklist that highlights key issues for nurses involved in caring for patients receiving immune checkpoint inhibitor therapy is presented in Appendix C.

**Appendix C:**
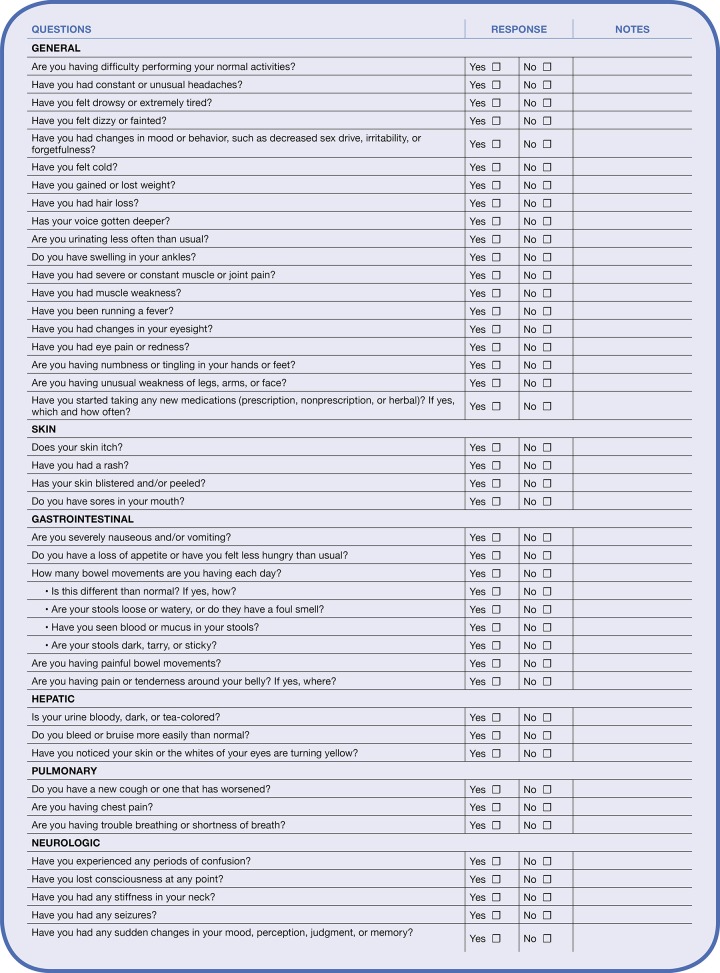
Appendix C Nurse’s Checklist for Immune Checkpoint Inhibitor Therapy

## MANAGING PATIENTS WITH IRAES: A PRACTICAL APPROACH

The following case studies illustrate the identification and management of irAEs with immune checkpoint inhibitor therapy and outline the role of advanced care providers, such as nurse practitioners and PAs, in identifying and managing these irAEs.

**Case 1: Nephritis and Rash**

A 54-year-old male with conjunctival *BRAF* wild-type melanoma metastatic to bilateral lungs and cervical lymph nodes received nivolumab and ipilimumab combination in an expanded-access program. A history of hypertension complicated by retinopathy and a history of seasonal erythematous rash were noted. Before initiation of therapy, the advanced care provider met with the patient and family for an educational session. Potential adverse effects of the regimen were reviewed, and the patient was alerted to symptoms he needed to report. An emergency contact phone number was provided. After the patient received his first dose of therapy, the advanced care provider initiated surveillance phone calls aimed at early detection of toxicities.

The patient developed grade 2 maculopapular rash after the first dose, which was managed by holding the patient’s second dose of treatment and same-day referral to dermatology for biopsy. Acute spongiotic and vesicular dermatitis with eosinophils were identified and treatment initiated with clobetasol 0.05% topical cream. Following resolution of an acute cutaneous response, he remained on a reduced dose of prophylactic clobetasol. The role of advanced care providers is essential during dermatologic evaluation and in identifying the need for timely referral to dermatology. After evaluation by dermatology service, the patient continued on close observation during clinic visits and phone surveillance calls for rebound signs and symptoms of rash while on topical steroid treatment.

Before the start of cycle 3, the patient’s metoprolol regimen (100 mg daily) for treatment of hypertension was changed to hydrochlorothiazide at 100/25 mg daily, which resulted in mild improvement in hypertension. Following dose 3, grade 2 asymptomatic creatinine elevation occurred (1.4 mg/dL) and was managed by an increase in oral hydration. As the creatinine level increased to 2.1 mg/dL 6 days later, the patient was instructed to begin oral prednisone at 60 mg daily and discontinue the angiotensin-receptor blocker. The patient was also referred to renal services for the evaluation of acute kidney injury. Creatinine returned to near baseline (0.9 mg/dL) within 3 days of prednisone initiation, and prednisone was tapered by 10 mg daily. Urinalysis revealed normal results, and the patient completed the fourth dose of therapy with continued close monitoring of renal function.

Following his first dose of nivolumab monotherapy, he developed grade 2 alanine aminotransferase elevation, which was managed by holding the following dose and treatment with prednisone at 90 mg daily, followed by a 3-week taper schedule. The patient’s laboratory evaluation returned to normal values, and repeated imaging revealed an excellent response to treatment, with substantially decreased bilateral pulmonary metastases; decrease in the size of the bilateral hilar, intraparotid, and cervical lymph nodes; and no new adenopathy.

This case demonstrates the importance of collecting a detailed and updated medical history to establish a baseline. The etiology of elevated creatinine in this case may have been due to autoimmune interstitial nephritis or may have been related to the initiation of angiotensin-receptor blocker therapy. A patient’s history can assist clinicians with the general direction for monitoring while on immunotherapy, and advanced health care providers such as nurse practitioners and PAs are well positioned to establish a baseline health history and exam and to conduct thorough clinical evaluations at each clinic visit.

Upon development of irAEs, referral to specialists (e.g., dermatology and renal service in this case) for early evaluation and intervention can assist in the initiation of therapy that will target symptoms and minimize compromise of patient safety. Although immune-related phenomena of cutaneous toxicities caused by immunotherapy are well known, autoimmune conditions such as nephritis are relatively rare and complex. Advanced health care providers play a critical role in educating and guiding patients in the recognition of AEs and in facilitating timely referrals to specialty services. 

**Case 2: Hypothyroidism, Pneumonitis, and Hemolytic Anemia**

A 71-year-old female with *BRAF* mutation–positive melanoma presented with metastases to the lungs, spleen, abdominal lymph nodes, sternum, right lateral sixth rib, and right ilium subsequent to disease progression on vemurafenib (Zelboraf). In a phase I study, she received 4 doses of combination nivolumab and ipilimumab over 12 weeks, followed by nivolumab monotherapy every 2 weeks. She experienced no toxicities and a partial response, with a 69% decrease in tumor burden after 12 weeks of combination therapy.

Grade 2, asymptomatic hypothyroidism (onset at day 15 of nivolumab monotherapy) was initially treated with levothyroxine at 25 µg while continuing nivolumab; the patient was later referred to endocrinology, and levothyroxine was increased to 100 µg. Grade 1 periorbital edema relating to hypothyroidism was noted on day 15 of monotherapy cycle 3, shortly after the increased dose of levothyroxine, and was closely observed without further adjustment in levothyroxine.

At the start of cycle 4, imaging revealed a continuing partial response, with an 85% decrease in tumor burden and grade 1 pleural effusions. A transthoracic echocardiogram ruled out potential cardiac etiology, and nivolumab monotherapy was withheld. Two weeks later, the patient had developed grade 1 nonproductive cough and a chest CT scan found bilateral pleural effusions and new ground-glass opacities demonstrating grade 1 pneumonitis. Nivolumab continued to be withheld, and the patient was referred for thoracic consultation. Surveillance with serial imaging was recommended.

On day 29 of cycle 5, the patient presented with a total bilirubin of 1.7 mg/dL, hemoglobin of 8.7 g/dL, rising lactate dehydrogenase of 1,208 U/L, grade 1 fatigue, and jaundice. She was referred for inpatient admission, and further workup was consistent with pernicious anemia. Treatment was initiated with intramuscular injection of vitamin B12 (1,000 µg daily) for 1 week, followed by successive weekly injections, and the patient was discharged.

At the start of cycle 7, the patient had multiple new and enlarging pulmonary nodules but resolution of pleural effusions. She was referred to interventional radiology for lung biopsy, and treatment with nivolumab was resumed. The biopsy revealed nondiagnostic parts of lung parenchyma with areas of chronic inflammation. Furthermore, the patient experienced a decline in hemoglobin, escalation of bilirubin and lactate dehydrogenase, and a positive super Coombs test. Nivolumab was withheld, and a course of prednisone was implemented.

The patient was tapered off prednisone during cycle 9 and found to have complete resolution of hemolytic anemia. Cycle 10 assessment imaging revealed resolution of grade 1 pneumonitis; however, dominant splenic lesion measurements increased. Monotherapy was resumed at the start of cycle 11, and the patient received her last nivolumab infusion on day 29 of cycle 11. An assessment scan on day 43 of cycle 11 revealed stability of the splenic lesion, but the patient experienced early signs of resumption of hemolytic anemia and underwent surgical resection and splenectomy. Response was a 94% decrease in tumor burden before surgical resection, with no evidence of disease after surgery.

This case demonstrates that collaboration among a multidisciplinary team is essential for the care of patients being treated with immunotherapy. The complexity of this case and development of numerous side effects while on therapy reveal the importance of vigilant monitoring, and advanced care providers are uniquely positioned to help establish an appropriate timeframe for AE monitoring and open communication among services.

Recommendations for identification and management of immune-related pneumonitis seen primarily with PD-1 blockade deserve special mention. Signs and symptoms include radiographic changes, new or worsening cough, chest pain, and shortness of breath (Bristol-Myers Squibb, 2015a). Patients should be monitored for signs and symptoms and evaluated with radiographic imaging for suspected pneumonitis. Corticosteroids should be administered at a dose of 1 to 2 mg/kg/day prednisone equivalents for grade 2 or greater pneumonitis, followed by a corticosteroid taper. Anti–PD-1 therapy should be permanently discontinued for severe (grade 3) or life-threatening (grade 4) pneumonitis and withheld until resolution for moderate (grade 2) pneumonitis (Bristol-Myers Squibb, 2016; Merck, 2015a).

Special considerations in the management of irAEs relate to certain patient populations. The safety of immune checkpoint inhibition in patients with underlying autoimmune disorders has not been evaluated in clinical trials, as patients with autoimmune conditions are typically excluded. Expert recommendations highlight the need for a careful risk/benefit analysis in such patients (Postow, 2015). Registrational trials of ipilimumab, nivolumab, and pembrolizumab monotherapy included sufficiently high numbers of elderly patients (aged ≥ 65 years) to indicate no overall differences in safety or efficacy, suggesting these patients may be treated as the general population (Bristol-Myers Squibb, 2015c, 2016; Merck, 2015a). Based on CheckMate 069 and 067, data also suggest similar outcomes for nivolumab and ipilimumab combination therapy in elderly patients (Postow et al., 2015; Larkin et al., 2015a).

## SUMMARY

Immune checkpoint blockade has emerged as a promising new treatment strategy, with three immune checkpoint inhibitor antibodies currently approved by the FDA for the treatment of metastatic melanoma as monotherapy (ipilimumab, pembrolizumab, and nivolumab) as well as in combination (nivolumab and ipilimumab). A clear understanding of the distinct immune-mediated safety profile of these agents is critical to their safe and appropriate use. The irAEs are well known, and several management algorithms, practical checklists, and tools have been established to aid patient management. Clinical data suggest that appropriate immunosuppressive treatment does not impair therapeutic efficacy, and most irAEs resolve with the use of immunomodulatory medications.

These novel therapies have opened a new avenue for antitumor response. However, we must recognize that patients who have been treated with immune checkpoint inhibitors may develop immune-related patterns of response that may deviate from Response Evaluation Criteria in Solid Tumors as a result of immune infiltration at tumor sites. Clinical and radiographic evaluation is imperative in determination of clinical benefit, and advanced health-care providers such as nurse practitioners and PAs are ideally placed to monitor, educate, and liaise with patients and the multidisciplinary team to facilitate early identification and intervention should irAEs occur, ensuring optimal management and patient outcome.

**Acknowledgments**

This project was funded in part through the NIH/NCI Cancer Center Support Grant P30 CA008748. Professional medical writing and editorial assistance was provided by Zenab Amin, PhD, and Cara Hunsberger at StemScientific, an Ashfield Company, funded by Bristol-Myers Squibb.
